# High-fat diet impairs glucose homeostasis by increased p16 beta-cell expression and alters glucose homeostasis of the progeny in a parental-sex dependent manner

**DOI:** 10.3389/fendo.2023.1246194

**Published:** 2023-10-09

**Authors:** Rene Escalona, Carlos Larqué, Daniela Cortes, Ricardo Vilchis, Emiliano Granados-Delgado, Abigail Sánchez, Guadalupe Sánchez-Bringas, Haydée Lugo-Martínez

**Affiliations:** Laboratory of Embryology and Genetics, Departamento de Embriología y Genética, Facultad de Medicina, Universidad Nacional Autónoma de México, Ciudad de México, Mexico

**Keywords:** intergenerational, obesity, adiposity, hyperinsulinemia, impaired-glucose tolerance, sexual dimorphism, parental-obesity

## Abstract

**Introduction:**

Obesity consists in the accumulation of adipose tissue accompanied by low grade chronic inflammation and is considered a pandemic disease. Recent studies have observed that obesity affects females and males in a sex-dependent manner. In addition, several works have demonstrated that parental obesity increases the risk to develop obesity, insulin resistance, diabetes, and reproductive disorders. Considering that intergenerational effects of obesity may occur in a sex-dependent manner, we studied male *Wistar* rat progeny (F1) obtained from mothers or fathers (F0) fed on a high-fat diet (HFD).

**Methods:**

Five-week-old female and male *Wistar* rats were fed on a HFD (with 60% of calories provided by fat) for 18 weeks (F0). At the end of the treatment, animals were mated with young rats to obtain their progeny (F1). After weaning, F1 animals were fed on standard chow until 18 weeks of age. Body weight gain, fasting plasma glucose, insulin and leptin levels, glucose tolerance, insulin sensitivity, and adiposity were evaluated. In addition, beta-cell expression of nuclear p16 was assessed by immunofluorescence.

**Results and conclusions:**

HFD altered plasma fasting glucose, insulin and leptin levels, glucose tolerance, adiposity, and beta-cell expression of p16 in F0 rats. Particularly, HFD showed sexual dimorphic effects on body weight gain and insulin sensitivity. Moreover, we observed that parental HFD feeding exerts parental-sex-specific metabolic impairment in the male progeny. Finally, parental metabolic dysfunction could be in part attributed to the increased beta-cell expression of p16; other mechanisms could be involved in the offspring glucose homeostasis.

## 1 Introduction

Obesity is a public health concern, according to the WHO. In the last 45 years its incidence has tripled around the world (1). Worldwide, it is estimated that 1.9 billion adults are obese or overweight. Excess of body weight is a significant risk factor for developing metabolic syndrome, type-2 diabetes mellitus (T2DM), cardiovascular diseases, among other ailments. Generally speaking, it is considered that obesity is the result of an imbalance between calorie intake and expenditure; however, there is ample evidence that involves multiple additional factors (2).

Furthermore, it has been considered that environmental factors might be involved in the development of obesity and T2DM. In particular the intrauterine environment has been suggested as a critical element associated with an increased BMI (3). The exposure to increased levels of nutrients, hormones and growth factors during critical windows of development, may program the metabolic health of the progeny. Epidemiological evidence has shown that maternal metabolic health correlates with that of its progeny; however, these findings might be confounded by the fact that both, the mother and the offspring, share the same obesogenic environment. However, animal experiments have shown that maternal obesity impairs energy homeostasis in its progeny (4). Although maternal obesity has been placed in the spotlight of the intergenerational transmission of obesity; there is growing evidence in support of the role of paternal metabolic health. It has been observed that diet-induced obese mice could sire offspring with impaired metabolic health for up to two generations suggesting that the metabolic state of each parent influences the metabolic health of the progeny, raising the issue of the impact of both obese parents (5). Additionally, these findings raise the concern of sexual dimorphism in the intergenerational transmission of obesity. Studies in humans had observed sex-specific effects of parental and grandparental nutrition in the development of diabetes mellitus (6). Finally, there are some experiments in mice that have shown a sex-specific inheritance of metabolic disturbances (7, 8).

Given the social, economic and scientific relevance of the obesity epidemic, it is worthwhile to investigate all the associated factors. In this paper we employed Wistar rats fed a high-fat diet to generate a parental obesity model (F0). These rats were mated with young non-obese rats to investigate the dimorphic effects of parental obesity in the metabolic health of the progeny (F1). In order to exclude environmental factors, F1 rats were fed normal chow, to determine exclusively the role of maternal or paternal obesity. We observed that parental obesity and metabolic disturbances have a sex-specific manner of inheritance. Thus, providing further evidence of additional factors involved in the increasing rate of obesity observed nowadays.

## 2 Materials and methods

### 2.1 Animals

Twenty one-day postnatal (p21) male and female Wistar rats were obtained from the animal facility of the Instituto de Fisiología Cellular, Universidad Autónoma de México (UNAM). All animals were kept in the vivarium of the Department of Embryology and Genetics according to the Internal Committee for Use and Care of Laboratory Animals (CICUAL) of the Facultad de Medicina, UNAM; and to the “International Guiding Principles for Biomedical Research Involving Animals”, Council for International Organizations of Medical Sciences 2010. The animals were maintained with *ad libitum* access to water and food and were housed under constant temperature (22°C) and lighting conditions, with a 12:12 h light-dark cycle, except during the mating period, when the cycle was modified to 14 h of lighting and 10 h of darkness.

### 2.2 Diet

After two weeks of acclimatization, animals were randomly separated in control (C) or high fat diet (HFD) group. Control group was fed with a standard commercial diet (Lab Diet 5001), with a caloric density of 4.1 Kcal/g. The high fat diet (HFD) was designed and manufactured in our laboratory. For every 100 grams of HFD, 49.5 g of pulverized pellets of standard chow were enriched with 17.75 g of olive oil (Borges ®), 17.75 g of lard (J.C. Forte), and 15 g of freeze-dried food grade egg albumin (Inova R) to balance the protein intake. The HFD had a caloric density of 5.82 Kcal/g with 60% of the calories coming from lipids.

### 2.3 Experimental design

#### 2.3.1 F0 generation

To obtain the F0 generation, 35 days-old rats were randomly separated into C and HFD groups. The feeding paradigm was as follows: during 18 weeks the groups of female (f), and male (m) rats were fed with standard diet (SD) as the control group; or with HFD. Body weight gain (g) and food intake (Kcal/24 hour) were recorded weekly on an individual basis. At the end of the experimental period, all groups of rats, C (f and m) and HFD (F and M), were divided into two subgroups. One of them was euthanized, while the other was used to breed the F1 generation.

#### 2.3.2 F1 generation

F0 animals were mated with lean and healthy animals of reproductive age. Once the mating period was over, F0 females (f and F) and males (m and M) were separated from their mates. The progeny obtained consisted of four groups: control males from Ctrl-fed mothers (fm), males from HFD-fed mothers (Fm), males from Ctrl-fed fathers (mm), and males from HFD-fed fathers (Mm). At birth all litters of the F1 generation were culled to 8 animals to ensure homogenous access to food during the lactation period (3 weeks). At weaning, all groups of the F1 generation were fed with the standard diet during 15 weeks.

### 2.4 F0 and F1 metabolic tests

One week before the end of the experimental period, an intraperitoneal glucose tolerance test (IPGTT) was performed with animals of both F0 and F1 generations. After an overnight fast, an intraperitoneal (IP) injection of a sterile dextrose (40%) solution at a dose of 2 g per kg of body weight. Three days after the IPGTT, an insulin tolerance test (ITT) was performed on overnight (12 hours) fasted rats using an IP injection of human recombinant insulin (Humulin®, Lilly) at a dose of 0.2IU per kg of body weight. For both tests, blood glucose (mg/dL) was determined at 0, 15, 30, 60, 90 and 120 minutes after glucose or insulin administration. Blood glucose determination for both tests was performed using a conventional glucometer (SD Check Gold®, Biosensor).

### 2.5 Blood samples and tissue collection.

At the end of the experimental period, both generations (F0 and F1) were euthanized by the administration of an IP injection of sodium pentobarbital (150 mg/kg). Immediately after loss of consciousness, the nasoanal and periabdominal length (cm) was measured for each animal. Blood samples were collected by cardiac puncture into EDTA containing microtubes. Blood was centrifuged to isolate plasma, which was stored at -70°C until analyzed. Following blood collection, the following adipose tissue depots were collected and weighed: supragonadal adipose tissue (SGAP), peripancreatic adipose tissue (PPAT), retroperitoneal adipose tissue (RPAT), and subcutaneous adipose tissue (ScAT). The weight of individual fat pads was reported as a proportion of total body mass (adiposity; % of body weight). The proportion of visceral adipose tissue (VAT) was estimated by the sum of SGAP, PPAT and RPAT.

### 2.6 Hormone and cytokine quantification

Plasma insulin, leptin, IL-6, IL-1β, and TNF-α were quantified by Milliplex immunoassay designed by Merck-Millipore (RADPKMAG-80K, MILLIPLEX MAP Rat Adipokine Pane), following the manufacturer instructions.

### 2.7 Quantification of plasma lipids

Quantification of plasma lipids was performed by the service platform of the Department of Pathology of the Facultad de Medicina Veterinaria y Zootecnia, UNAM. The technique used for detection and quantification was UV-visible spectrophotometry.

### 2.8 Pancreatic islets characterization

Pancreatic tissue was fixed in a PBS solution of 4% paraformaldehyde at 4°C overnight. After fixation, the tissue was processed for paraffin embedding, 5 µm sections were obtained and placed on gelatin-coated glass slides. Tissue sections were deparaffinized in xylol and rehydrated in gradual series of ethanol. In order to assess the proportion of senescent beta cells, a double immunofluorescence was performed. Antigen retrieval was done with 0.1M citrate buffer pH 6.0 in a pressure cooker at 90°C for 15 min. Following antigen retrieval, unspecific binding was blocked with 2% BSA, 2% NGS, and 0.5% Triton-X for 1 hour at RT. Beta cells were identified with a rabbit anti-insulin polyclonal antibody (PAA448Ra01, Cloud-Clone Corp, 1:2000),; additionally, senescent cells were labeled with a mouse anti-p16 monoclonal antibody (sc-1661, 1:50, Santa Cruz Biotechnology). Primary antibodies were incubated at RT during 48 hours; following this, the sections were incubated with a goat anti-mouse IgG coupled to Alexa 647 (1:400, 115-605-166, Jackson Immunoresearch), and goat anti-rabbit IgG coupled to Alexa 488 (111-545-003, 1:800, Jackson Immunoresearch). Nuclei were stained with DAPI (sc-3598, Santa Cruz Biotechnology); finally, the sections were mounted in a MOWIOL solution and coverslipped. Images were acquired with a Zeiss LSM-880 confocal microscope, and analyzed using FIJI software (Fiji Is Just ImageJ). At least 15 islets comprising 3 different regions separated 1 mm apart were analyzed for each animal.

### 2.9 Statistical analysis

All data were analyzed using OriginPro 2016 data analysis and graphing software, and R package version 4.1.2 (2021–11–01). It was determined whether the data showed a normal distribution. A Student’s T-test was used to compare the F0 generation. In case of F1 rats, ‘*n*’ represents the number of rats from individual litters. Data from the F1 generation were analyzed separately to compare the influence of parental sex, diet, or the interaction of both, using a two-way ANOVA. Specific comparisons were made using a *post-hoc* Tukey test. Data are presented as mean values ± standard error of the mean (SEM). *p* values ≤ 0.05 were considered significant. F and *p* values for each variable with significant influence of parental sex, diet or their interaction are shown in **Supplementary Table.**

## 3 Results

### 3.1 Characterization of maternal and paternal obesity

**Figure 1:**
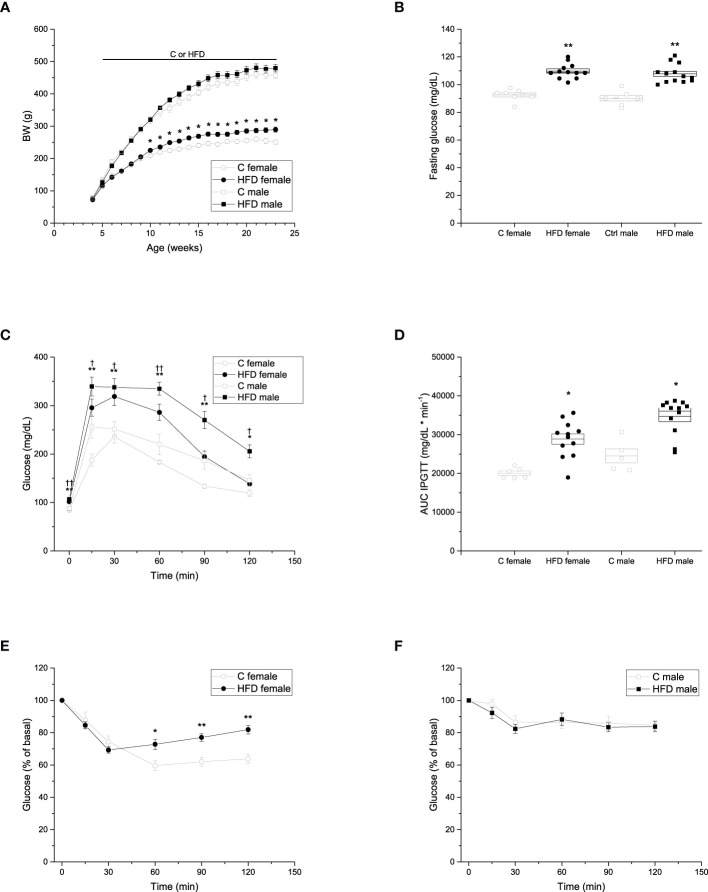
Metabolic characterization of the F0 generation. **(A)** Weekly body weight gain of F0 males (C n =7; HFD n =13) and females (C n =8; HFD n =12) during the 18 weeks of the experimental period. **(B)** Blood fasting glucose of F0 males (C n =7; HFD n =13) and females (C n =8; HFD n =12). **(C)** Intraperitoneal glucose tolerance test (IPGTT) of F0 males (C n =5; HFD n =12) and females (C n = 6; HFD n =12). **(D)** Area under the curve of the IPGTT. **(E)** Insulin tolerance test (ITT) of F0 females (C n = 7; HFD n =12) and males (C n =7; HFD n =13). Points in **(A, C, E, F)** represent mean ± SEM. Points in **(B, D)** represent individual animals, boxes represent SEM. */† represent significance against C, single and double scripts represent *p* < 0.05 and < 0.01, respectively.

To evaluate the effects of HFD administration for 18 weeks in female and male rats, we registered body weight gain weekly. We observed that body weight of HFD female (289.4 ± 7.8 g) rats was higher than that of control female (250.7 ± 6.3 g) rats from week 10 of age. However, no difference was observed in body weight of HFD male (479.3 ± 11.4 g) rats compared to control male (457.2 ± 10.0 g) rats (**Figure 1A**).

In addition, fasting glucose, IPGTT and ITT were performed to assess glucose homeostasis after 18 weeks of HFD administration. Fasting glucose levels of both female and male HFD groups were higher compared to those of control groups (**Figure 1B**) (C female = 92.5 ± 4.01 mg/dL; HFD female = 109.8 ± 5.39 mg/dL; C male = 90.1 ± 5.24 mg/dL; HFD male = 107.8 ± 6.66 mg/dL). Both female and male rats on a HFD showed glucose intolerance compared to the control groups, with higher levels of glucose throughout the test (C female = 20040.8 ± 552.48 mg/dL * min^-1^; HFD female 28865.8 ± 1354.72 mg/dL * min^-1^; C male = 24538.0 ± 1803.7 mg/dL * min^-1^; HFD male = 34713.7 ± 1342.3 mg/dL * min^-1^) (**Figures 1C, D**). Moreover, glucose levels of both HFD female and male rats did not return to basal levels at the end of the experiment (**Figure 1C**). Furthermore, HFD female rats (9299 ± 238% of basal glucose * min^-1^) showed impaired insulin sensitivity when compared to control female rats (7840 ± 695% of basal glucose * min^-1^), whereas no significant difference in insulin sensitivity was observed between male groups (**Figures 1E, F**).

**Figure 2:**
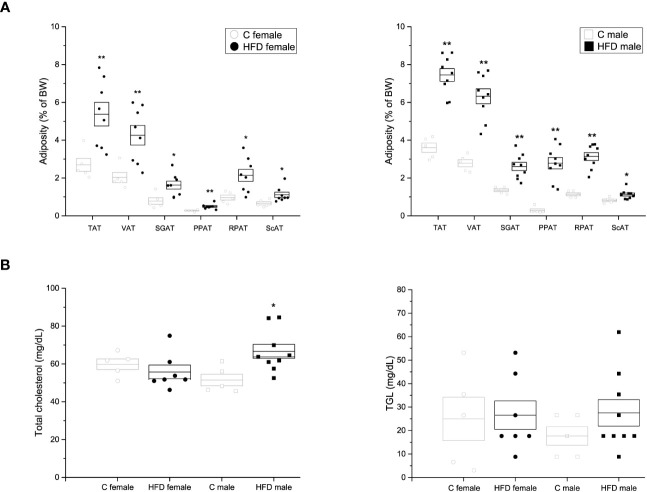
Adiposity and lipid quantification of the F0 generation. **(A)** Weight of adipose tissue depots as a percentage of body weight in F0 females (left; C n = 4; HFD n = 8) and males (right; C n = 5; HFD n = 9). Total adipose tissue (TAT), visceral adipose tissue (VAT), supragonadal adipose tissue (SGAP), peripancreatic adipose tissue (PPAT), retroperitoneal adipose tissue (RPAT), and subcutaneous adipose tissue (ScAT). **(B)** Total blood cholesterol (left) and triacylglycerols (TGL, right) of F0 females (C n = 5; HFD n = 7) and males (C n = 5; HFD n = 9). Points represent individual animals, boxes represent SEM. * and ** represent significance against C, *p* < 0.05 and < 0.01, respectively.

After 18 weeks of HFD administration, various fat pads were quantified to evaluate adiposity of animals. It was observed that female and male rats on a HFD showed an increase in the weight and body percentage (HFD female: TAT = 5.37 ± 0.62%, VAT = 4.26 ± 0.52%, SCAT = 1.11 ± 0.14%, RPAT = 2.14 ± 0.32%, SGAT = 1.62 ± 0.21%, PPAT = 0.49 ± 0.04%; HFD male: TAT = 7.45 ± 0.337%; VAT = 6.32 ± 0.396%; SCAT = 1.12 ± 0.082%; RPAT = 3.14 ± 0.207%; SGAT = 2.61 ± 0.216%; PPAT = 2.78 ± 0.299%) of fat pads, when compared to rats fed on a control diet (C female: TAT= 2.69 ± 0.34%, VAT = 2.03 ± 0.27%, SCAT = 0.66 ± 0.08%, RPAT =0.97 ± 0.13%, SGAT = 1.36 ± 0.08%, PPAT = 0.29 ± 0.08%; C male: TAT = 3.60 ± 0.246%; VAT = 2.78 ± 0.19%; SCAT = 0.82 ± 0.066%; RPAT = 1.13 ± 0.078%; SGAT = 1.36 ± 0.079%; PPAT = 0.28 ± 0.078%) (**Figure 2A**). Additionally, HFD-fed male (66.63 ± 3.71 mg/dL) rats exhibited an increase in plasma levels of total cholesterol when compared to control rats (51.52 ± 3.08 mg/dL *p* = 0.018) (**Figure 2B**). However, no significant difference in total cholesterol levels was observed between female groups (HFD female = 55.75 ± 3.59 mg/dL VS C female = 59.78 ± 2.78 mg/dL), nor in TG (HFD female = 26.55 ± 6.1 mg/dL VS C female = 38.94 ± 6.0 mg/dL; and HFD male = 27.53 ± 5.6 mg/dL VS C male = 17.7 ± 3.95 mg/dL) plasma levels was observed between groups (**Figure 2B**).

**Figure 3:**
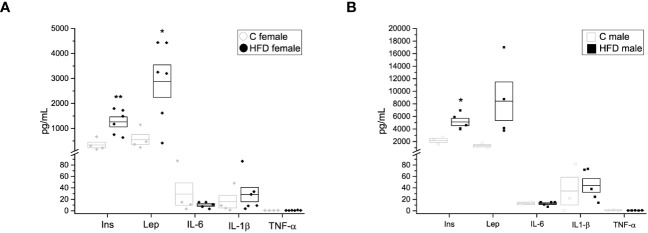
Hormone and cytokine quantification of the F0 generation. **(A)** Insulin (Ins), leptin (Lep), interleukin 6 (IL-6), interleukin 1β (IL-1 β) and tumor necrosis factor α (TNF-α) in F0 females (C female n = 4; HFD female n = 6), and **(B)** males (C male n = 3; HFD male n = 5). Points represent individual animals, boxes represent SEM. * and ** represent significance against control, *p* < 0.05 and < 0.01, respectively.

In addition, plasma levels of insulin, leptin and other adipokines were assessed. Female rats on a HFD exhibited higher levels of plasma insulin (338.6 ± 113.46 pg/mL) and leptin (2892.2 ± 651.21 pg/mL) when compared to the female control group (**Figure 3A**) (Ins = 338.6 ± 113.46, Lep = 556.3 ± 204.04 pg/mL), while male rats on HFD showed increased plasma levels of insulin (5107.2 ± 574.04 pg/mL) compared to the control group (2174.7 ± 327.31 pg/mL) (**Figure 3B**). Nevertheless, no significant differences were observed in plasma levels of IL-6, IL-1β nor TNF-α between HFD groups and control groups (HFD female: IL-6 = 10.27 ± 2.272 pg/mL, IL-1β = 28.318 ± 12.641 pg/mL, TNF-α = 0.792 ± 0.1469 pg/mL VS C female: IL-6 = 29.15 ± 19.55 pg/mL, IL-1β = 15.86 ± 10.96 pg/mL, TNF-α = 0.661 ± 0.0742 pg/mL; HFD male: IL-6 = 12.619 ± 1.578 pg/mL, IL-1b = 44.314 ± 12.1292 pg/mL, TNF-a = 0.7015 ± 0.8513 pg/mL VS C male: Lep = 1328.1 ± 200.6 pg/mL, IL-6 = 12.979 ± 2.02 pg/mL, IL-1b = 34.52 ± 24.315, TNF-a = 1.008 ± 0.3347 pg/mL) (**Figures 3A, B**).

**Figure 4:**
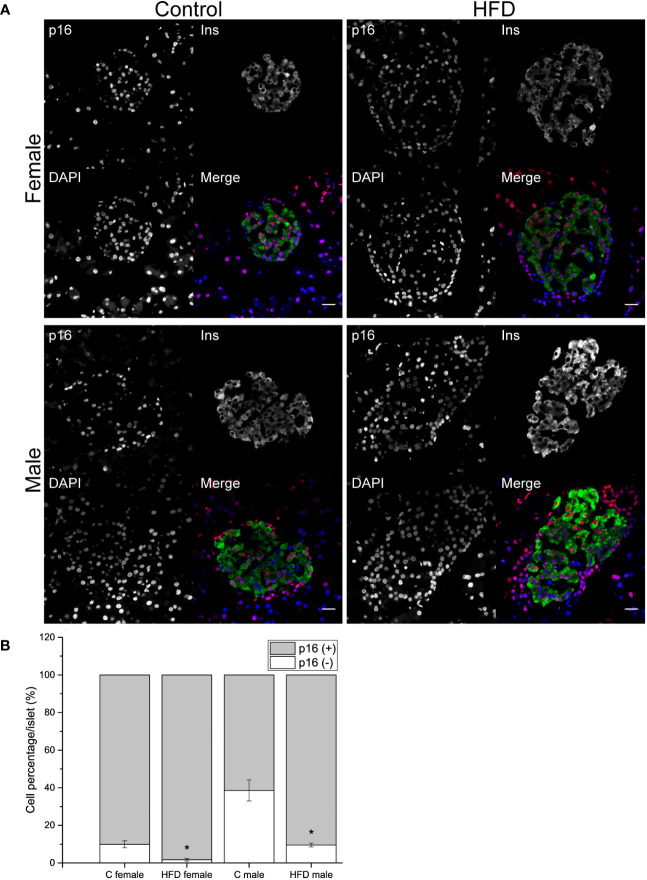
Pancreatic islets characterization of the F0 generation. **(A)** Representative islets of F0 male (C male n = 3; HFD male n = 4) and females (C female n = 5; HFD female n = 3) from C and HFD groups, sections were stained for insulin (green), p16 (red) and DAPI (blue); scale bar = 20 µm. **(B)** Proportion of senescent (p16^+^/ins^+^) and non-senescent (p16^-^/ins^+^) beta-cells in all F0 groups. Bars represent the mean SEM, * represent significance against control *p* < 0.05.

In order to explore if the administration of HFD induced a change in the proportion of p16 expressing and non-expressing beta cells, pancreatic sections were analyzed. An increase in the proportion of p16 (+) beta cells was observed both in female (p16^+^ cells = 98.3 ± 0.75%) and male (p16^+^ cells = 90.4 ± 1.02%) rats fed on HFD compared to control diet (C female: p16^+^ cells = 90.1 ± 1.9%; C male: p16^+^ cells = 61.4 ± 5.59%). On the other hand, the proportion of non-expressing p16 beta cells decreased in animals fed on HFD compared to the control groups (**Figures 4A, B**).

### 3.2 Maternal- and paternal-obesity intergenerational metabolic effects on the offspring

Male descendants were obtained from rats fed on control diet and HFD for 18 weeks. In case of the HFD-female rats, diet was suspended at delivery and control diet was administered during lactation to avoid any dietary effect.

**Figure 5:**
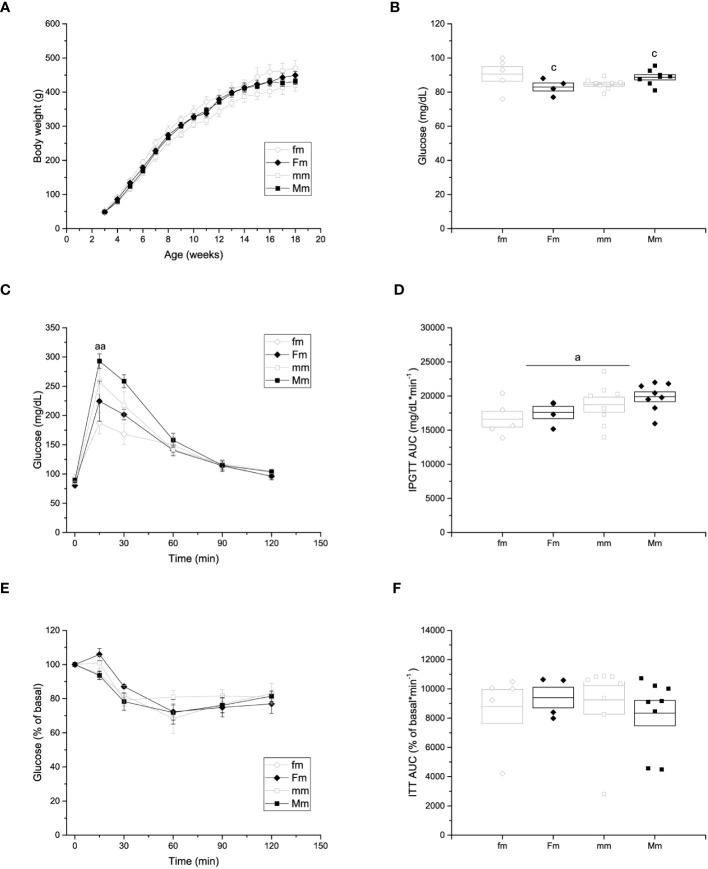
Metabolic characterization of F1 males. **(A)** Weekly body weight gain of F1 males sired by C females (fm; n = 9), C males (mm; n = 12); and by HFD females (Fm; n= 7) and HFD males (Mm; n = 16). **(B)** Blood fasting glucose (fm n = 9; mm = 12; Fm = 7; Mm = 12). **(C)** Left, intraperitoneal glucose tolerance test (IPGTT; fm n = 9; mm = 11; Fm = 7; Mm = 10). **(D)** Area under the curve of the IPGTT. **(E)** Insulin tolerance test (ITT); fm n=9; mm= 12; Fm= 7; Mm= 23). **(F)** Area under the curve of the ITT. Points in **(A, C, E)** represent mean ± SEM. Points in **(B, D)** represent individual animals, boxes represent SEM. (a) indicates significant influence of parental sex, (b) indicates significant influence of parental diet, and (c) indicates a significant influence of the interaction of both factors. Single and double scripts represent *p* < 0.05 and < 0.01, respectively.

No differences were observed in the body weight of male offspring of HFD-fed female (449.07 ± 10.99 g) or male (431.15 ± 9.09 g) rats compared to male offspring obtained from control-fed (fm = 470.15 ± 22.66 g; and mm = 419.83 ± 16.36 g, respectively) animals (**Figure 5A**). Significant effects of interaction of parental sex and diet were observed on fasting glucose levels (fm = 90.6 ± 4.2 mg/dL; Fm = 83 ± 2.34 mg/dL; mm = 84.6 ± 1.1; and Mm = 88.7 ± 1.6 mg/dL) (**Figure 5B**). In addition, effects of parental sex were observed on glucose levels at 15 (fm = 187.2 ± 18.46 mg/dL; Fm = 224.5 ± 34.5 mg/dL; mm = 257.5 ± 20.67 mg/dL; Mm = 293 ± 12.52 mg/dL) minutes of the IPGTT. Nevertheless, no effects were observed over the glucose levels after 30, 60, 90 nor 120 minutes of the IPGTT (**Figure 5C**), and plasma glucose returned near to fasting levels after 120 minutes in all the male F1 groups Furthermore, significant effect of parental sex was found on glucose tolerance in the IPGTT (fm = 16609.6 ± 1151.52 mg/dL * min^-1^; Fm = 17587.6 ± 895.56 mg/dL * min^-1^; mm = 18724.4 ± 1098.89 mg/dL * min^-1^; Mm = 19891.5 ± 720.60 mg/dL * min^-1^) (**Figure 5D**). Finally, no effects of parental sex, diet, or their interaction was detected on insulin sensitivity in any F1 male group (**Figures 5E, F**).

**Figure 6:**
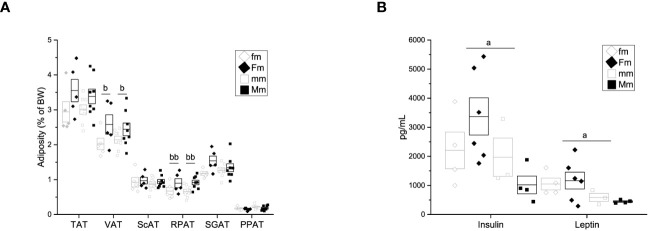
Adiposity, hormone and cytokine quantification of F1 males. **(A)** Weight of adipose tissue depots as a percentage of body weight (fm n = 4; mm = 4; Fm = 4; Mm = 6). Total adipose tissue (TAT), visceral adipose tissue (VAT), supragonadal adipose tissue (SGAP), peripancreatic adipose tissue (PPAT), retroperitoneal adipose tissue (RPAT), and subcutaneous adipose tissue (ScAT). **(B)** Insulin (Ins), leptin (Lep), interleukin 6 (IL-6), interleukin 1β (IL-1 β) and tumor necrosis factor α (TNF-α) (fm n = 3; mm = 3; Fm = 6; Mm = 7). (a) indicates significant influence of parental sex, (b) indicates significant influence of parental diet, and (c) indicates a significant influence of the interaction of both factors. Single and double scripts represent *p* < 0.05 and < 0.01, respectively.

Despite there was no difference in body weight among male-descendant groups, we assessed adiposity to further explore intergenerational obesity effects on adipose tissue. A significant effect of parental diet on the percentage of visceral adipose tissue (fm = 2.01 ± 0.164% of BW; Fm = 2.58 ± 0.276% of BW; mm = 2.14 ± 0.101% of BW; Mm = 2.44 ± 0.181% of BW) at the expense of the retroperitoneal fat depot (fm = 0.674 ± 0.105% of BW, Fm = 0.896 ± 0.128% of BW; mm = 0.661 ± 0.060% of BW; Mm = 0.918 ± 0.065% of BW) was observed (**Figure 6A**).

Additionally, significant effect of parental sex was found on insulin (fm = 2203.4 ± 627.02 pg/mL, Fm = 3371.34 ± 639.60 pg/mL, mm = 1969.39 ± 660.07, Mm = 1019.33 ± 305.26 pg/mL), and leptin (fm = 1048.02 ± 201.19 pg/mL, Fm = 1165.30 ± 290.61 pg/mL, mm = 587.34 ± 140.37 pg/mL, Mm = 445.00 ± 27.15 pg/mL) plasma levels (**Figure 6B**). Plasma levels of IL6, IL1-β, and TNF-α were assessed; however, no difference was observed in any F1 male group (data not shown).

**Figure 7:**
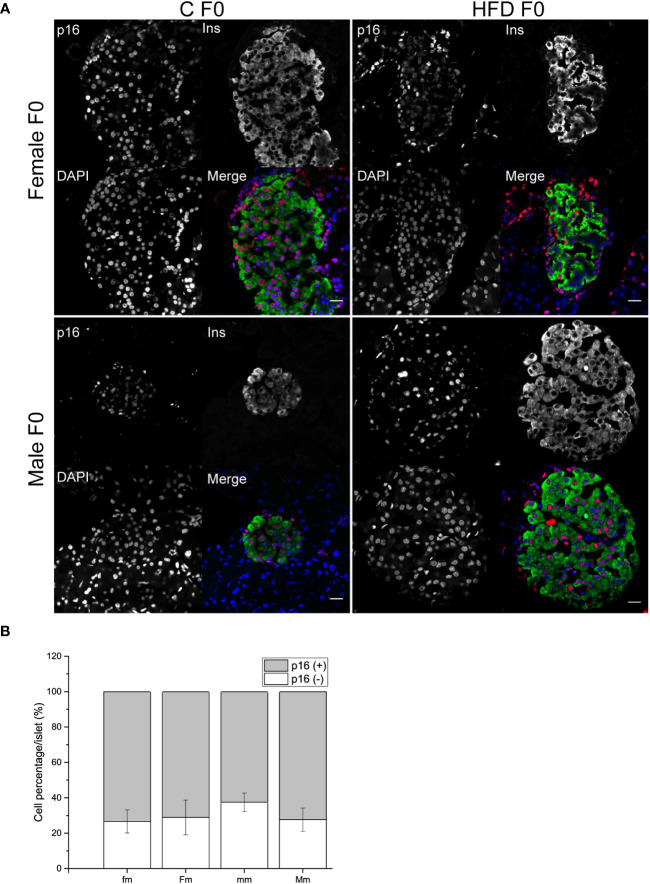
Pancreatic islets characterization of F1 males. **(A)** Representative islets of F1 males sired from C and HFD parents (fm n = 4; mm = 3; Fm = 3; Mm = 4). Sections were stained for insulin (green), p16 (red) and DAPI (blue); scale bar = 20 µm. **(B)** Proportion of senescent (p16^+^/ins^+^) and non-senescent (p16^-^/ins^+^) beta cells F1 males. Bars represent mean ± SEM. (a) indicates significant influence of parental sex, (b) indicates significant influence of parental diet, and (c) indicates a significant influence of the interaction of both factors. Single and double scripts represent *p* < 0.05 and < 0.01, respectively.

Finally, we decided to further explore if the intergenerational effects of obesity could increase pancreatic beta-cell p16 expression as a possible pathophysiological mechanism of impaired fasting glucose, hyperinsulinemia, and glucose intolerance observed in male progeny. However, no effect of parental sex, diet or interaction was observed on the proportion of p16^+^ or p16^-^ beta cells (**Figures 7A, B**).

## 4 Discussion

Given the significance of the obesity pandemic, it is of great importance to evaluate all the potential metabolic impairments that parental obesity can inflict on the upcoming generations. Thus, the aim of this study was to assess the metabolic alterations in the progeny of high-fat fed males and females. In particular, we were interested in whether these possible disturbances could be transmitted in a sex-dependent manner.

In the F0 generation we found that the administration of a HFD induced an expansion of adipose tissue. Consistent with our results, it has been shown that HFD based on lard or olive oil lead to obesity and increased adiposity in Wistar rats (9). It is worth noting that in our model, rather than a significant increase in body weight, an increase of visceral adipose tissue was observed in HFD fed animals. Visceral fat has been associated with a greater release of cytokines, fatty acids and triacyclglycerols (10). In humans, it has been linked to poorer metabolic and health outcomes. Thus, the increased adiposity and metabolic disturbances observed in HFD fed animals make a reasonably good model of obesity. Interestingly, olive oil has been widely considered to have multiple health benefits. This contrasts with our results, in which a HFD based on lard and olive oil induced increased adiposity and glucose intolerance among other metabolic disturbances. There are reports that show that excessive fat consumption can override the beneficial effects often attributed to olive oil. A previous study reported that a HFD in which 60% of total calories were provided by olive oil induced liver steatosis, increased body weight, altered OGTT in mice (11). Furthermore, an olive oil based HFD has been shown to produce similar results to sunflower oil (12). These results suggest that the total amount of fat-derived calories could be the principal factor involved in the induction of metabolic alterations.

While the impact of maternal obesity over the metabolic fate of its offspring has been extensively studied, the possible consequences of paternal obesity have received relatively less attention. However, there is evidence in humans that has shown that paternal metabolic status has a direct impact over BMI and systolic blood pressure of its offspring during adolescence (13). Furthermore, there is evidence in rodent models that paternal obesity impairs the physiology of its descendants. For example, F1 male offspring obtained from obese mice display a body weight and adiposity similar to that of males sired by lean mice, but exhibit a significant decrease in sperm count and motility (14). Moreover, another report shows that in Sprague-Dawley rats paternal obesity induces beta cell dysfunction only in female descendants, through non-genetic transmission (15). Contrary to our results, they observed impaired insulin secretion and glucose homeostasis in F1 females. One possible explanation could be the composition of the HFD; the HFD diet employed in that study was composed primarily of saturated fats, while our diet contains a relatively higher proportion of monounsaturated fatty acids. Finally, in agreement with our results, it has been shown that in mice, paternal administration of HFD has sex-related consequences to the next generation (5). In the same study, the authors identified changes in testicular levels of mRNA and miRNA involved in ROS production, lipid metabolism and embryo development. Furthermore, metabolic changes in testicular tissue have been suggested to be involved in the paternal transmission of metabolic and reproductive dysfunction; in particular the fatty-acid content of sperm cells (16). Thus, the possible mechanisms involved in paternal transmission of metabolic disorders could be attributed to changes in lipid and RNA composition of sperms exposed to the obesogenic environment.

On the other hand, several studies have demonstrated that maternal obesity increases the risk for development of obesity, T2DM, and cardiovascular diseases (17). Studies in humans have shown that male offspring (4-7 years old) of mothers exposed to a high eating index during pregnancy, exhibited increased levels of glucose, insulin and other adipokines (18). In addition, other studies have observed a potential synergistic relationship between maternal overweight and high glucose levels, that may increase the risk of childhood overweight (19). Maternal obesity has also been associated with a diabetic phenotype which includes glucose intolerance, hyperinsulinemia, and insulin resistance in adult offspring, particularly in males (20). In the present study, metabolic traits such as fasting glucose, glucose tolerance, and insulin plasma levels were more affected in male descendants of HFD-fed animals than in female descendants of HFD-fed rats. Some mechanisms involved in the sexual dimorphism of inheritance of metabolic traits have been proposed and include chromosomic content, genomic imprinting, hormone content, and different developmental processes. For example, association between the enzyme content and the sex chromosome-bearing of sperm has been observed in bulls (16, 21). Moreover, sex hormones are main determinants of multiple sexually dimorphic traits. Additionally, sex-specific and parent of origin-inheritance of metabolic traits is a complex process that involves multiple elements. While many studies attribute this phenomenon to epigenetic inheritance, most of them are based on correlational analyses. Therefore, further experiments are needed in order to properly define the mechanisms involved.

Multiple studies in animal models of obesity have demonstrated that long-term metabolic impairment could lead to pancreatic beta-cell dysfunction through diverse mechanisms including endoplasmic reticulum stress, unfolded protein response, or apoptosis (22). Additionally, accumulation of senescent beta cells associated with obesity or impairment in glucose metabolism has been observed (23). In addition, the removal of p16 expression, has been shown to delay age-related beta cell dysfunction (24). In addition, it has been shown that beta cells expressing senescence-related markers such as p16 exhibit increased insulin secretion during incubation at low-glucose conditions (25). This work demonstrates that HFD-fed female and male rats accumulate an increased proportion of p16+ beta cells compared to control-fed animals, this may be related to the basal hyperinsulinemia observed. It is worth noting that the observed proportion of p16+ beta cells is higher to what has been observed in mice (26). However, our results are similar to other works that observed approximately 70% of p16+ beta cells in 5-month old Wistar rats without sex specification (27).

Nevertheless, in the present study, no difference in the proportion of p16+ beta cells was observed in the descendants of HFD-fed animals compared to the control group. However, considering that cell senescence is a complex process that involves multiple pathways and molecular markers, we cannot fully exclude senescence as one of the mechanisms involved in the intergenerational disorders observed. Basal hyperinsulinemia, and insulin resistance have been observed in the offspring of HFD-exposed male mice (28). A previous study analyzed pancreatic islet function in the offspring of obese female mice. Female offspring exhibited increased glucose stimulated insulin secretion (GSIS), and mitochondrial respiration rate (29), suggesting that not only cellular senescence, but other mechanisms could be involved in the metabolic impairment of the progeny an obese parent. In addition, other studies in mice have observed that the offspring exposed to maternal obesity may show an age- and diet-dependent progressive loss of beta cell function and impairment of glucose homeostasis (30).

Thus, further exploration related to the metabolic programming of beta cell characteristics such as GSIS, ion channel activity, metabolic function, and immaturity markers could contribute to determine the precise mechanisms underlining hyperinsulinemia and glucose intolerance observed. Moreover, administration of a HFD to the offspring of obese animals may potentiate any subtle metabolic impairment that could increase the susceptibility to develop impaired fasting glucose, glucose intolerance, metabolic syndrome, or T2DM.

In summary, we observed that parental HFD feeding exerts parental-sex-specific metabolic impairment in the male progeny. Although, parental metabolic dysfunction could be in part attributed to the increased beta-cell expression of p16; other mechanisms could be involved in the offspring glucose homeostasis. Therefore, further investigation focused on the mechanisms involved in the metabolic alterations observed in the progeny is required.

## Data availability statement

The original contributions presented in the study are included in the article/**Supplementary Material**. Further inquiries can be directed to the corresponding author.

## Ethics statement

The animal study was approved by Comité Interno para el Cuidado de Animales de Laboratorio, Facultad de Medicina, UNAM. The study was conducted in accordance with the local legislation and institutional requirements.

## Author contributions

RE, CL, and HL-M contributed to conception and design of the study, animal care, organize the database, performed statistical analysis, wrote sections of the manuscript. DC, EG-D, RV, and AS contributed to data acquisition, animal care. GS-B contributed to animal care. All authors contributed to the article and approved the submitted version.
