# Supplementary figures and images for: High-fat diet impairs glucose homeostasis by increased p16 beta-cell expression and alters glucose homeostasis of the progeny in a parental-sex dependent manner
Source: Front Endocrinol (Lausanne). 2023 Oct 9;14:1246194. doi: 10.3389/fendo.2023.1246194 (PMC10591070; doi:10.3389/fendo.2023.1246194)

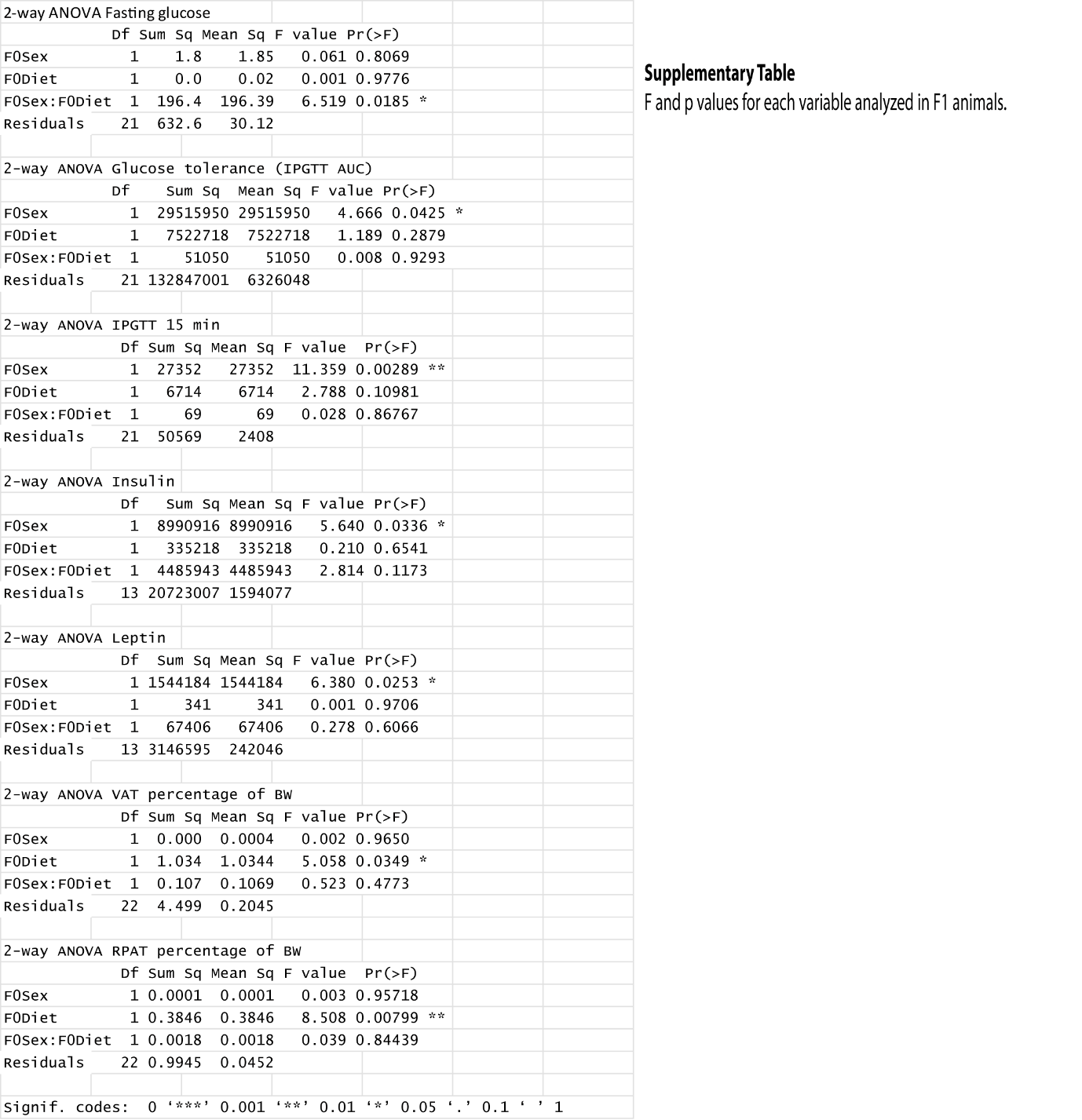

Supplement: Supplementary file 1 [file Table_1.docx]
